# A microdeletion at Xq22.2 implicates a glycine receptor *GLRA4* involved in intellectual disability, behavioral problems and craniofacial anomalies

**DOI:** 10.1186/s12883-016-0642-z

**Published:** 2016-08-09

**Authors:** Jonathan D. J. Labonne, Tyler D. Graves, Yiping Shen, Julie R. Jones, Il-Keun Kong, Lawrence C. Layman, Hyung-Goo Kim

**Affiliations:** 1 Department of Obstetrics & Gynecology, Section of Reproductive Endocrinology, Infertility & Genetics, Medical College of Georgia, Augusta University, 1120 15th Street, Augusta, GA 30912 USA; 2 Department of Neuroscience and Regenerative Medicine, Medical College of Georgia, Augusta University, 1120 15th Street, Augusta, GA 30912 USA; 3 Department of Laboratory Medicine, Boston Children’s Hospital, Harvard Medical School, Boston, MA 02115 USA; 4 Greenwood Genetic Center, Greenwood, SC 29646 USA; 5 Department of Animal Science, Division of Applied Life Science (BK21plus), Institute of Agriculture and Life Science, Gyeongsang National University, Jinju, Gyeongsangnam-do South Korea; 6 Neuroscience Program, Medical College of Georgia, Augusta University, Augusta, GA 30912 USA

**Keywords:** *GLRA4*, Xq22.2, Pseudogene, Intellectual disability, Behavioral problems, Craniofacial anomalies, Microdeletion

## Abstract

**Background:**

Among the 21 annotated genes at Xq22.2, *PLP1* is the only known gene involved in Xq22.2 microdeletion and microduplication syndromes with intellectual disability. Using an atypical microdeletion, which does not encompass *PLP1*, we implicate a novel gene *GLRA4* involved in intellectual disability, behavioral problems and craniofacial anomalies.

**Case presentation:**

We report a female patient (DGDP084) with a *de novo* Xq22.2 microdeletion of at least 110 kb presenting with intellectual disability, motor delay, behavioral problems and craniofacial anomalies. While her phenotypic features such as cognitive impairment and motor delay show overlap with Pelizaeus-Merzbacher disease (PMD) caused by *PLP1* mutations at Xq22.2, this gene is not included in our patient’s microdeletion and is not dysregulated by a position effect. Because the microdeletion encompasses only three genes, *GLRA4*, *MORF4L2* and *TCEAL1*, we investigated their expression levels in various tissues by RT-qPCR and found that all three genes were highly expressed in whole human brain, fetal brain, cerebellum and hippocampus. When we examined the transcript levels of *GLRA4*, *MORF4L2* as well as *TCEAL1* in DGDP084′s family, however, only *GLRA4* transcripts were reduced in the female patient compared to her healthy mother. This suggests that *GLRA4* is the plausible candidate gene for cognitive impairment, behavioral problems and craniofacial anomalies observed in DGDP084. Importantly, glycine receptors mediate inhibitory synaptic transmission in the brain stem as well as the spinal cord, and are known to be involved in syndromic intellectual disability.

**Conclusion:**

We hypothesize that *GLRA4* is involved in intellectual disability, behavioral problems and craniofacial anomalies as the second gene identified for X-linked syndromic intellectual disability at Xq22.2. Additional point mutations or intragenic deletions of *GLRA4* as well as functional studies are needed to further validate our hypothesis.

**Electronic supplementary material:**

The online version of this article (doi:10.1186/s12883-016-0642-z) contains supplementary material, which is available to authorized users.

## Background

Intellectual disability (ID) is a genetically heterogeneous condition with 2 % of the population estimated to be impacted by this disorder [1, 2]. Such impairment of cognitive functions places a high burden on families as well as society making ID an important problem in medicine [3]. Notably, X-linked intellectual disability (XLID) is approximately 4 times more prevalent than autosomal forms [4]. Of the ~1,098 genes residing on the X chromosome [5], a large number play a role in neurological functions [4, 6], and some of these genes are highly expressed in the brain [7]. More than 100 ID genes residing on the X-chromosome have been identified, and it is thought that more XLID genes are yet to be discovered, particularly in X-linked families where the genes map to new loci [2, 8–10].

Being approximately 1 Mb in size, chromosomal segment Xq22.2 contains at least 21 annotated genes including *TCEAL1* (MIM 300237), *MORF4L2* (MIM 300409), *PLP1* (MIM 300401) and an apparent pseudogene (*GLRA4*) [11]. Among these genes, *PLP1* mutations have been previously shown to be associated with the neurological disorder Pelizaeus-Merzbacher disease (MIM 312080) characterized by intellectual disability, nystagmus, ataxia, spasticity, and microcephaly. Brain MRI revealed macrogyria, dysmyelination, and increased paramagnetic signal which is indicative of iron deposition [12, 13]. Furthermore, *PLP1* is also involved in Xq22.2 microdeletion and microduplication syndromes [8].

The apparent human pseudogene glycine receptor α4-subunit (*GLRA4*) lacks the 4th transmembrane domain (TM4) located at the C-terminal end of the protein [11, 14, 15]. Glycine receptors (GLRs) are important in mediating inhibitory neurotransmission in the brain [14, 16, 17]. However, the role played by *GLRA4* in humans is unclear because the absence of the TM4 domain is expected to be damaging to GLRA4 protein function [13]. Pseudogenes have been long considered as silent relics residing in the genome as neutral sequences with no biological roles [18, 19]. However, over the past decade, evidence is mounting that pseudogenes have biological functions and may play a role in health and disease [19–22]. For instance, post-transcriptional silencing of *HMGA1* (MIM 600701) by its pseudogene (*HMGA1-p*) leads to insulin resistance and type 2 diabetes [22]. Similarly, pseudogene *PTENP1* has been shown to exert a growth-suppressive role by regulating the transcript levels of the tumor suppressor gene *PTEN* (MIM 601728) [21].

Here, we report an 11-year-old female patient presenting with intellectual disability, behavioral problems and craniofacial anomalies. We performed microarray and identified an apparent ~105 Kb microdeletion at Xq22.2. The genomic deletion encompasses the genes *TCEAL1* (MIM 300237), *MORF4L2* (MIM 300409) and an apparent pseudogene *GLRA4*. We performed qPCR to refine the locations of the proximal as well as the distal deletion breakpoints. The myelin proteolipid protein-coding gene (*PLP1*) resides ~ 46 kb distal to the microdeletion. We examined the transcript levels of *GLRA4*, *TCEAL1*, *MORF4L2* as well as *PLP1* (MIM 300401) in the patient and family members. The protein levels of PLP1 were also examined by Western blot to determine whether *PLP1* is dysregulated by a position effect. We assayed the level of transcripts of *GLRA4*, *MORF4L2*, and *TCEAL1* in the brain and other tissues. We hypothesize that *GLRA4* is a novel candidate gene for XLID and is likely involved in the clinical features observed in our patient including cognitive impairment, behavioral problems and speech delay.

## Case presentation

The proband (DGDP084) is an 11-year-old girl born after normal pregnancy and delivery. At birth, she weighed 2.83 kg. She passed her neonatal hearing screen test and did not require admission to the special care unit. At around 8 weeks, the infant started manifesting nystagmus. An MRI turned out to be normal. At 8 months, her parents started to have concerns about her motor skills. She was not yet sitting or weight bearing. She continued to display jerky eye movement and showed some reluctance to elevate her eyes. Upon examination at 9 months, development of her gross motor skills was found to be delayed. At 15 months, her gross motor skills were determined to be at the 9-month-level. Her parents also noted that she was slow to develop movement patterns, particularly involving rotation. At that time, the infant was described as a happy, smiling little girl.

When examined at 20 months, she was still showing developmental delay. She was not yet crawling, and used a lot of jargon with no clear words. She was able to roll and enjoyed clapping and waving goodbye. At 2 years and 1 month she had a brief generalized tonic clonic seizure associated with fever. As a 2 year and 3-month old girl, she was able to recognize her name and understand the names of familiar adults. She was crawling efficiently and enjoyed turning pages of her books. She was able to bring a spoon to her mouth, but required her parents’ assistance for loading. A thyroid function test performed at 2 years and 8 months did not detect any abnormalities. Her development at that time was quite significantly delayed in many areas and she was functioning at around the 12-month level. She walked with a wide spaced ataxic gait and displayed some stereotypical hand movements. The girl also had episodes where she would stare and look blankly for approximately 10 s. She displayed some of the physical and behavioral features of Angelman syndrome. She has had extensive testing of genetic changes normally seen in children with Angelman syndrome. This has excluded a maternally inherited deletion, uniparental disomy, CNV analysis, abnormal methylation at 15q11-q13, or a mutation in *UBE3A* gene as causative. As some children with *MECP2* deletions have an Angelman-like phenotype, gene testing of *MECP2* (MIM 300005) was also done, which was also negative. Mowat-Wilson syndrome (MIM 235730), clinically similar to Angelman syndrome, is caused by mutations of *SIP1* (*aka ZEB2*, MIM 605802). Mutation screening of this gene was negative. Although Angelman syndrome patients show specific EEG patterns, an EEG showed no specific or paroxysmal abnormality in DGDP084. This suggests that she was not suffering from Angelman syndrome.

**Fig. 1:**
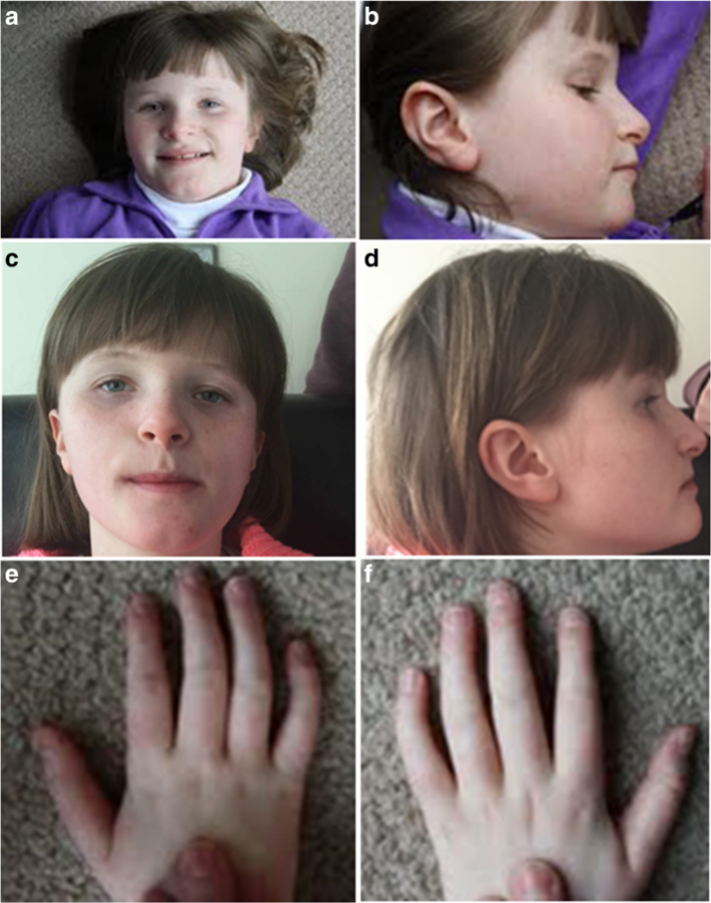
Facial and hand pictures of patient DGDP084. Facial picture showing a broad face with prominent chin at (**a**) 9 years and 2 months (**c**) 12 years and 2 months. Lateral facial view of the patient showing low-set ears at (**b**) 9 years and 2 months (**d**) 12 years and 2 months. Hands and finger shape (**e** and **f**) showing fifth finger clinodactyly as well as mild tapering of fingers at 9 years and 2 months

At 3 years and 4 months, the patient had a head circumference at 49.5 cm (50th centile). She had deep blue, lightly pigmented irides and displayed frontal bossing as well as a flat occiput. The patient also had a prominent chin (Fig. 1a) and displayed fifth finger clinodactyly (Fig. 1e and f). When she was examined at 4 years-1-month, her parents revealed that their daughter’s sleep patterns were disturbed. She would wake up in the middle of the night and start banging on her window. At 4 years-7-months, she displayed global developmental delay, hypermetropic astigmatism, and minor jerky eye movement. Her height was at the 25th centile and weight on the 75th centile. At that time, she was also enrolled in special schooling. She had several episodes of significant abdominal pain associated with constipation and was being treated with Movicol.

As a 5-year-old girl, the patient displayed significant learning difficulties. She was also experiencing intermittent episodes of distress during which she was making straining noises. These episodes occurred approximately every month or two, lasting up to one week. During those episodes, she often sat in an unusual position with her legs folded underneath, her head held back and in a dream-like state. She was also diagnosed with glycosuria. At that time, the patient had a head circumference of 55.5 cm (between the 9th and 25th centile), weighed 19.4 kg (50th centile), and her height was at 107.4 cm (25th centile). Her sleep patterns were still variable despite being treated with Melatonin. She continued to have an ataxic wide-based gait and displayed stereotypic arm movements with hand flapping. She also had a tendency to walk in a side-to-side stepping instead of forward stepping. Microarray analysis revealed a deletion at Xq22.2.

When examined as a 6-year-old girl, she weighed 18.7 kg (9th-25th centile), and her height was 110.1 cm (9th-25th centile). She displayed poor coordination, and her speech had not yet developed. The girl was diagnosed with Type 1 diabetes, and a heart murmur was also detected. She continued to have problems with constipation and was taking Chloral hydrate. Her parents mentioned that her problematic sleep patterns were becoming a concern as the banging noises were disturbing neighbors. The repeated banging also left marks on her wrist, and she was also eating non-food items. She was dependent on her parents for all her personal care and was also enrolled in occupational therapy. While still a 6-year old girl, she had chicken pox, which was mild in nature. She continued to have unsettled nights as a 7-year-old girl and at that time the abdominal discomfort was thought to be presumably responsible. A summary of DGDP084 clinical features is provided in Table 1.

**Table 1:** Clinical features of DGDP084 compared with other patients having small-sized microdeletions within the Xq22.2 interval

Clinical features	DGDP084	Yamamoto	Torisu	Inoue 2002	Inoue 2002	Inoue 2002	Matsufuji 2013
		2014 Pt5	2012	Pt 1	Pt 2	Pt 3	Pt II-1
	F	F	M	M	M	M	M
Intellectual disability	+	+	+	+	+	+	+
Speech delay	+	+	+	NS	+	+	+
Motor delay	+	+	+	+	+	+	+
Impaired social skills	+	+	-	NS	+	NS	+
Sleep disturbance	+	+	NS	NS	NS	NS	NS
Behavioral problems	+	+	NS	NS	NS	NS	NS
Seizures	+	-	NS	NS	-	NS	-
Craniofacial anomalies	+	+	+	NS	NS	NS	NS
Tapering fingers & clinodactyly	+	-	NS	NS	NS	NS	NS
Brain MRI – delayed myelination	-	+	+	+	+	NS	+
Cardiac anomalies	+	-	NS	NS	NS	NS	NS
Gastrointestinal problems	+	-	NS	NS	NS	+	+

*NS* not stated, *Pt* patient, *F* represents female while *M* denotes male

Family history is unremarkable except for the father, paternal grandmother and a maternal cousin. The patient’s father has Type I diabetes, while the maternal cousin displays cerebral palsy. The paternal grandmother had a number of stillborn babies.

## Methods

Lymphoblastoid cell lines were made from blood samples obtained from patient DGDP084 and her parents using a density gradient centrifugation method following Nishimoto et al. [23]. Genomic DNA was isolated from blood samples using a standard phenol-chloroform protocol with minor modifications. The extracted genomic DNA from the patient’s blood was examined on a comparative genomic hybridization array (aCGH) to detect pathogenic copy number variations (CNVs) as described [24]. The X chromosome inactivation (XCI) pattern using genomic DNA from patient DGDP084 was determined by PCR analysis of a polymorphic CAG repeat in the first exon of the androgen receptor (*AR*) gene. Methylation of sites close to this short tandem repeat has been demonstrated to correlate with X chromosome inactivation [25]. In this assay, amplification of the *AR* gene both before and after digestion with the methylation-sensitive *HpaII* restriction enzyme was used to determine the methylation status of the maternal and paternal X chromosome. XCI degree threshold patterns are classified as random (XCI < 80 %), moderately skewed (80 % ≤ XCI ≤ 90 %), and highly skewed (>90 %) [26].

Primers targeting the putative proximal and distal deletion breakpoints at Xq22.2 were designed for qPCR. We also made primers against exonic sequences of *GLRA4*, *TCEAL1*, *MORF4L2*, and *PLP1* for qPCR as well as RT-qPCR (Additional file 1: Table S1). Total RNA was extracted from cell lines using the RNeasy Plus Mini kit (Qiagen) following the manufacturer’s instructions. RT-qPCR was performed using total RNA (Clontech) from whole human brain, fetal brain, cerebellum, cerebral cortex, hippocampus, kidney, liver, lung, heart and skeletal muscle. cDNA was synthesized from 1 μg of total RNA using the RevertAid First cDNA Synthesis Kit (Thermo Scientific). Real-Time PCR was carried out using 2 μl cDNA, 2.5 μM primer and 10 μl FastStart DNA Green Master (Roche) in a 20 μl reaction volume. Samples were run in triplicates and standard errors were calculated from 2–3 independent experiments. The ∆∆ct method was used to determine copy number of the loci of interest as well as relative transcript levels of genes of interests. The beta-2-microglobulin (MIM 109700) gene was used for data normalization. Numerical means and standard deviations for qPCR and RT-qPCR are provided in Additional file 1: Table S2 and S3, respectively. Protein was isolated from cell lines derived from patient DGDP084 and family members. Anti-PLP1 antibody (Abcam) targeting amino acids109-128 was used to detect PLP1 protein expression levels. A dilution of 1:1000 was used for the primary antibody, while the secondary anti-rabbit antibody (Thermo Scientific) was diluted 1:1000. Detection was carried out using the Amersham™ ECL™ Western Blotting Analysis System (GE Healthcare).

## Results

### Microarray analysis reveals a microdeletion in patient DGDP084 at Xq22.2

**Fig. 2:**
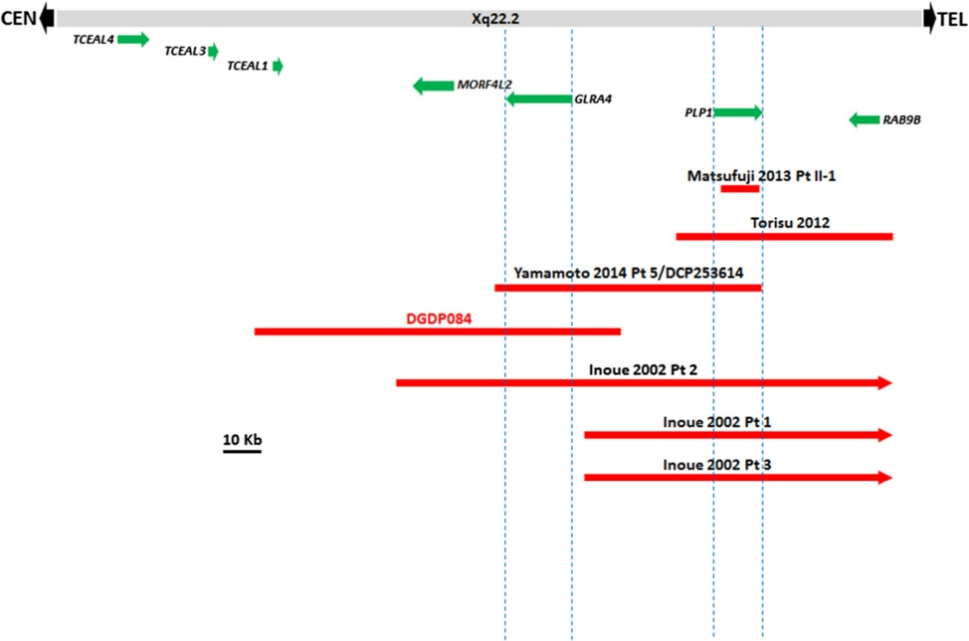
Comparative deletion mapping at Xq22.2 showing the size of the microdeletion in DGDP084 relative to six other patients. *GLRA4* is completely deleted in three patients. All six genomic deletions involve *PLP1*, except the microdeletion in DGDP084. The distal breakpoints of the three microdeletions reported by Inoue et al. [29] extend well beyond the *RAB9B* gene. However, the distal breakpoint in the patient described in Torisu et al. [28] is located immediately distal to *RAB9B*

A 105 kb minimal microdeletion at Xq22.2 (chrX: 102,882,657-102,987,229, hg 19) was detected by microarray analysis with a putative maximal deletion determined to be ~ 145 kb (chrX:102,857,905-103,002,957, hg 19). The deleted genomic region contains only three genes namely, *MORF4L2*, *GLRA4*, and *TCEAL1* (Fig. 2 and Table 2).

**Table 2:** Genes deleted in DGDP084

Gene	Gene symbol	OMIM#	Remarks
Glycine receptor, alpha 4 subunit	*GLRA4*	-	Glycine receptors mediate neurotransmission in the spinal cord and brain stem. Human *GLRA4* is considered as a pseudogene and has a stop codon before the predicted transmembrane domain (exon 9) [11, 36].
Mortality factor 4 like 2	*MORF4L2*	300409	May be implicated in cellular senescence like 2 [52]. The mouse homolog (*MgrX*) is not essential for growth and development [37]
Transcription Elongation Factor A-Like 1	*TCEAL1*	300237	Nuclear phosphoprotein having 48 % Elongation homology to transcription factor SII factor A-like-1 [53].

### Microdeletion occurred as a *de novo* event

**Fig. 3:**
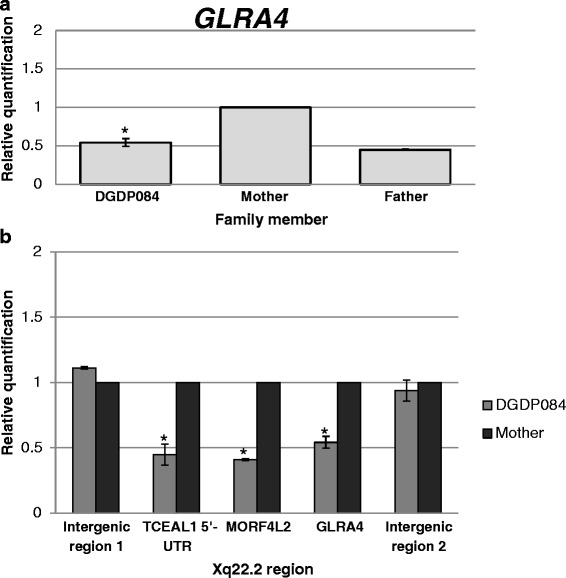
Refining deletion breakpoints in DGDP084. **a** qPCR assays confirmed the Xq22.2 microdeletion in DGDP084. *GLRA4* copy number in DGDP084 was compared to her mother as a gender control. The patient’s mother was normal possessing two copies of *GLRA4*, while the father and the affected female DGDP084 have only one *GLRA4* allele on the chromosome X. **b** The copy number of genes and loci in DGDP084 at Xq22.2 were compared to her mother. The *TCEAL1* gene was found to be completely deleted with the proximal deletion breakpoint lying between *TCEAL1* and *TCEAL3*. The distal deletion breakpoint was located in the non-genic region between *GLRA4* and *PLP1*. Asterisks denote differences that are statistically significant

qPCR assays using genomic DNA and primers targeting *GLRA4* confirmed the Xq22.2 microdeletion in female DGDP084 and also revealed that the chromosomal rearrangement occurred as a *de novo* event. As expected, the patient’s healthy mother possesses two copies of *GLRA4*, while the healthy father has only one copy (Fig. 3a). According to qPCR analysis the size of the microdeletion is at least 110 kb and does not extend into the proximal genes, *TCEAL3* and *PLP1*. The centromeric deletion breakpoint resides in the intergenic region 1 between *TCEAL1* and *TCEAL3*, while the telomeric breakpoint was found to reside in the intergenic region 2 between *GLRA4* and *PLP1* (Figs. 2 and 3b).

### Transcript levels of *GLRA4* are reduced in patient DGDP084

**Fig. 4:**
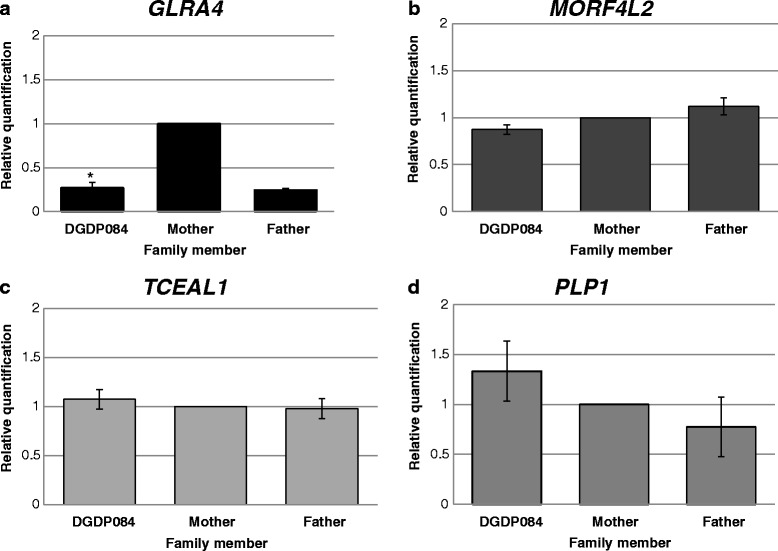
Assaying transcript levels of *GLRA4*, *MORF4L2*, *TCEAL1*, and *PLP1* by RT-qPCR. Transcript levels of the aforementioned genes in DGDP084 were compared to the patient’s mother as a gender control. **a** *GLRA4* transcripts were reduced in the female DGDP084 to the level of her healthy father compared to her healthy mother. **b** *MORF4L2* and **c** *TCEAL1* transcripts levels were similar in all family members. **d** The level of transcripts of *PLP1* was slightly increased in DGDP084, while a moderate reduction was noted in the patient’s father. However, both changes observed were statistically insignificant. Please see western blot of PLP1. Asterisks denote differences that are statistically significant

Analysis of *GLRA4* transcript levels by RT-qPCR revealed low *GLRA4* expression in DGDP084 girl similar to her healthy father, but much lower than her mother (Fig. 4a). The transcript levels of *MORF4L2* in DGDP084 were statistically not different to her parents (Fig. 4b). *TCEAL1* transcript level was consistent across all family members (Fig. 4c). The levels of *PLP1* transcripts in DGDP084 were statistically not different to her healthy mother (Fig. 4d).

### Random X-chromosome inactivation in DGDP084

X-inactivation studies revealed an inactivation ratio of 77:23. Since inactivation ratios of less than 80:20 are considered random patterns, this suggests a random inactivation of chromosome X in this microdeletion female. The X-chromosome with the Xq22.2 microdeletion in DGDP084 is not preferentially inactivated.

### *GLRA4*, *MORF4L2* and *TCEAL1* are highly expressed in the brain

**Fig. 5:**
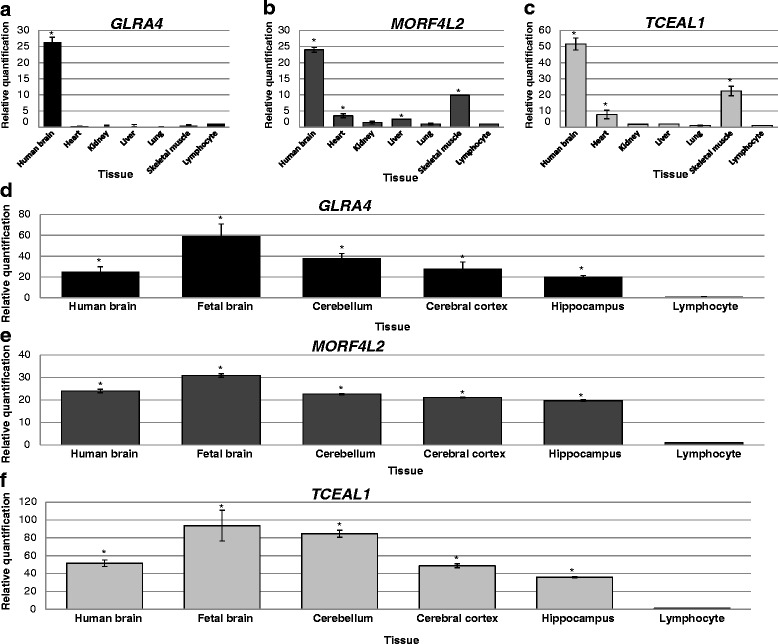
Transcript levels of *GLRA4*, *MORF4L2* and *TCEAL1* in the brain and other tissues. All three X-linked genes are highly expressed in the brain relative to lymphocytes. Low levels of transcripts were detected in the heart, kidney, liver and lung. Fetal brain and cerebellum showed the highest expression of *GLRA4*, *MORF4L2* and *TCEAL1*. High level of transcripts was also detected in the hippocampus compared to lymphocytes

Transcripts of *GLRA4* were ~25-fold higher in whole human brain relative to lymphocytes. Little or no *GLRA4* transcripts were detected in the heart, kidney, liver, lung, and skeletal muscle (Fig. 5a). The level of *MORF4L2* transcripts was ~24-fold higher in the brain, and 10-fold higher in skeletal muscle relative to lymphocytes (Fig. 5b). Very low levels of *MORF4L2* transcripts were detected in other tissues assayed. Transcript levels of *TCEAL1* were extremely high in the brain (~50-fold) compared to lymphocytes (Fig. 5c). High levels of *TCEAL1* transcripts (20-fold) were also detected in skeletal muscle relative to lymphocytes. Kidney, liver and lung expressed very low levels of *TCEAL1* transcripts.

Transcripts of *GLRA4*, *MORF4L2* and *TCEAL1* were abundantly expressed in fetal brain, cerebellum, cerebral cortex and hippocampus (Fig. 5d-f). The highest transcript levels were detected in fetal brain where an ~60-fold, ~30-fold, ~90-fold higher level of transcripts were detected for *GLRA4*, *MORF4L2* and *TCEAL1*, respectively relative to lymphocytes.

### Western blot shows that *PLP1* is not dysregulated by position effect

**Fig. 6:**
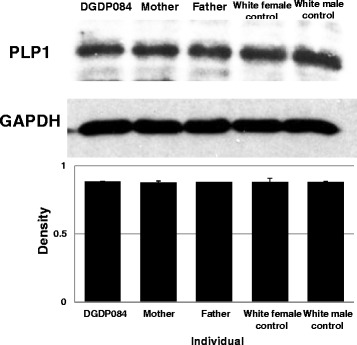
Western blot showing PLP1 expression levels in DGDP084 and her family members. The protein level of PLP1 in the patient was similar to her parents as well as the white female and male controls. GAPDH protein levels were used as a reference for densitometry analysis

By densitometry, the PLP1 protein was found to be expressed at the same levels in DGDP084, her parents and the two controls as normalized to GAPDH (Fig. 6). No gender difference in PLP1 protein expression was observed across the individuals assayed. We did not detect GLRA4 protein in lymphocytes but low levels were expressed in fibroblasts (data not shown).

### Comparative deletion mapping at Xq22.2

We performed comparative deletion mapping with DGDP084 and six additional informative microdeletions at Xq22.2 (Fig. 2). Patient II-1 of Matsufuji et al. [27] has a 33-kb microdeletion spanning *PLP1* only. The CNV in patient 5 of Yamamoto et al. [8] encompasses *GLRA4* and *PLP1*only, while the microdeletion in the patient of Torisu et al. [28] includes *PLP1* and *RAB9B* only. Patient 2 of Inoue et al. [29] has a microdeletion involving at least *MORF4L2*, *GLRA4*, *PLP1*, *RAB1* whereas his patients 1 and 3 of Inoue et al., [29] have CNVs encompassing at least *PLP1* and *RAB9B*. The deletion breakpoints of the three patients reported in Inoue et al. [29] have not been clearly defined. It is possible that *GLRA4* is dysregulated in patients 1 and 3 due to a position effect, in case their centromeric breakpoints do not affect *GLRA4*. Our patient DGDP084 without the deletion of *PLP1* displays clinical features including intellectual disability, speech delay, and craniofacial anomalies which are also present in the patient of Torisu as a result of *PLP1* deletion (Table 1). Interestingly, patients displayed a wider range of clinical features when *GLRA4* is included in the Xq22.2 microdeletion. In the case of DGDP084, the phenotype was more severe compared to other patients.

### The fourth transmembrane domain (TM4) is conserved among several species except human

**Fig. 7:**
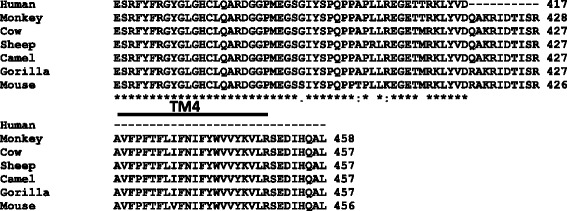
*GLRA4* C-terminal end amino acid alignment across seven species including mouse, human, gorilla, monkey, camel, sheep and cow. The last 90 amino acids are displayed along with the location of the TM4 domain. Human GLRA4 is the only species missing the conserved TM4 domain

Amino acid residues of GLRA4 are evolutionarily conserved throughout the seven mammalian species. Only human GLRA4 (NP_001019623.2) shows an absence of 40 amino acids at the C-terminal region, part of which codes for the fourth transmembrane domain TM4 (Fig. 7) [11]. The amino acid sequence of the TM4 domain is highly conserved among other species.

## Discussion

The Xq22.2 region harbors *PLP1* causative for Pelizaeus-Merzbacher disease characterized by cognitive impairment, severe spasticity, hypotonia, nystagmus and ataxia [12]. Most of the CNVs in this interval include *PLP1* and neighboring genes, suggesting the occurrence of microdeletion and microduplication syndromes at Xq22.2 with phenotypic features including intellectual disability (ID), behavioral abnormalities, and hypotonia [8]. The patient DGDP084 investigated in the present study displays intellectual disability, behavioral problems, and craniofacial anomalies. She has a *de novo* Xq22.2 microdeletion of at least 110 Kb encompassing only three annotated genes namely, *MORF4L2* and *TCEAL1* as well as an apparent human pseudogene *GLRA4* [11, 14, 15]. Both microarray and qPCR (Fig. 3) show that *PLP1* located immediately distal to *GLRA4* is not deleted (Fig. 2). Moreover, DGDP084 did not display anomalies in myelination, and the brain scan (MRI) performed was normal, making it highly unlikely that *PLP1* is the underlying cause of the clinical features. This is substantiated by Western blot showing similar levels of PLP1 expression in DGDP084 and her parents (Fig. 6), suggesting this gene is not dysregulated by a position effect. The cytogenetic band of Xq22.2 with a large interval of 1 Mb containing at least 21 annotated genes suggests the presence of one or more XLID genes in this region. So far, *PLP1* is the only gene known to be involved in XLID, and importantly, this is the first case of microdeletion with syndromic intellectual disability at Xq22.2 without involvement of *PLP1*. Conspicuously, the transcript levels of *GLRA4* were significantly reduced in DGDP084 compared to her healthy mother and this is consistent with the random X-inactivation result suggesting chromosome X with the Xq22.2 microdeletion is not preferentially inactivated. The higher dosage of *GLRA4* in the healthy mother compared to her healthy husband suggests that females express higher levels of *GLRA4* compared to males by either escaping X-inactivation or incomplete silencing of the inactive X chromosome occurred in this family. Notably, *MORF4L2* and *TCEAL1* transcripts were expressed at similar levels in the patient and her parents. The transcript levels of *GLRA4*, *MORF4L2* and *TCEAL1* were significantly higher in the whole human brain and fetal brain compared to other tissues such as heart, liver and kidney (Fig. 5a-c). High level of transcripts of all three genes were detected in various areas of the brain including the cerebellum and hippocampus (Fig. 5d-f), regions of the brain known to be associated with learning and memory [30]. The reduction of *GLRA4* transcript levels in DGDP084 in the family coupled with its expression pattern in the brain suggest that *GLRA4* is the plausible candidate gene for the clinical features observed in DGDP084 including intellectual disability, behavioral abnormalities, and craniofacial anomalies. Interestingly, the phenotype observed as a result of the microdeletion in DGDP084 shows overlap with the clinical features produced as a result of *PLP1* disruption including cognitive impairment and motor delay [8, 31] suggesting that both genes, physically located in close proximity, may participate in similar molecular function.

Comparable levels of *TCEAL1* and *MORF4L2* transcripts were detected in DGDP084 and her parents (Fig. 4b and c). This means that even if their high level of expression in the human brain and skeletal muscle (Fig. 5b and c) suggests a role in neurological and motor function, these genes may be excluded as contributing to cognitive impairment, behavioral abnormalities and craniofacial anomalies. Additional point mutations or intragenic deletions of *GLRA4* and functional studies are required to support our hypothesis. It is interesting to note that *TCEAL1* has been shown to interact with *PTPN11* [32], the causative gene for Noonan syndrome characterized by short stature, craniofacial anomalies, pectus deformities, webbed neck and sometimes ID [33, 34]. Moreover, *Mrgx*, the mouse orthologue of *MORF4L2* (aka *MRGX*, MIM 300409) has been shown to interact with *HNRNPK* [35], a gene known to be associated cognitive impairment [36]. However, knockout of *Mgrx* does not affect mouse development as the mice remain healthy and fertile [37].

Glycine receptor chloride channels (GLR) are pentameric proteins belonging to the Cys-loop family of ligand-gated ion channel receptors [14]. A total of five glycine receptor chloride channel subunit genes exist in humans, and these channels are usually composed of homo- or hetero-pentamers arranged around a central ion-conducting pore [38]. Functional homomeric α-GLRs have been shown to be assembled in the human embryonic kidney HEK-293 cell line [39], and their activation mechanisms have been studied in rat [40]. Most GLRs are assembled from four α-subunits (*GLRA1*, *GLRA2*, *GLRA3* and *GLRA4*) and one β-subunit (*GLRB*) [15, 41, 42]. Each subunit has an extracellular domain containing the neurotransmitter binding site, and a transmembrane domain comprising four transmembrane α-helices (TM1-TM4) connected by flexible loops [43]. The α subunits show a high degree of amino acid identity [44] indicating that they evolved by gene duplication [16, 45]. Being a transmembrane protein, GLRs selectively allow passage of Cl^-^ ions when glycine binds at specific sites on the receptor surface [41, 46]. GLRs are important in mediating inhibitory neurotransmission in a number of regions of the brain including mammalian brainstem and spinal cord where they are widely expressed [14, 16, 17]. Through electron microscopy, it has been shown that in rats GLRs are tightly packed around presynaptic terminals at central synapses [41, 46]. The functional importance of GLRs and the observed patterns of expression of *GLRA4* in the brain are consistent with a role in cognitive deficits and behavioral abnormalities observed in patient DGDP084.

Previously, it has been shown that mutations in the α1 subunit of glycine receptor (*GLRA1*) are responsible for the neurological disorder hyperekplexia (MIM 149400) characterized by muscle rigidity originating from the CNS as well as an exaggerated startle response [47]. Sudden muscle contractions are often observed in infancy and can sometimes lead to death possibly due to apnea or aspiration [48, 49]. Interestingly, a male patient possessing both missense and splice site mutations in *GLRB*, a β-subunit of the pentameric GLRs, has been reported to display hyperekplexia [50]. This shows that subtle alterations in the structure of GLRs can affect functioning of this ligand-gated ion channel in the brain. Our patient DGDP084 does not have hyperekplexia and it is possible that the cognitive impairment and unusual behaviors displayed by DGDP084 such as banging on windows and furniture may be due to reduced *GLRA4* expression in the brain disrupting the composition of heteromeric GLRs. It is interesting to note that patients with hyperekplexia display delay in speech acquisition and ID which usually normalize in infancy [43, 51].

In humans, the role of *GLRA4* in the functioning of GLRs is unclear because of the stop codon in exon 9 upstream of the predicted TM4 domain common in the three other human α subunits [11]. The sequence of each of the three GLR genes (α1- α3; *GLRA1*, *GLRA2*, *GLRA3*) encodes four TM domains which act as important barriers between the ion permeation pathway and the apolar areas of the lipid bilayer [41]. The absence of the TM4 domain in GLRA4 is thought to produce an inactive protein [11, 14, 15]. Interestingly, a recent study has shown that GLRA1 subunits with a recessive mutation p.E375X truncating upstream of TM4 domain are incorporated into functional pentametric GLRs and retain glycine sensitivity [43]. We propose the human GLRA4 protein lacking TM4 is not a pseudogene, but is required for the assembly and stability of some heteromeric GLRs. This is also substantiated by high levels of *GLRA4* transcripts detected in various part of the human brain including the cerebellum and hippocampus. Furthermore, we cannot exclude the possibility that *GLRA4* transcripts may also play a regulatory role in the brain by modulating transcription of other GLR genes through RNAi due to its high sequence homology with *GLRA1*, *GLRA2* and *GLRA3*.

Pseudogenes have previously been shown to have regulatory functions [20–22] and may play a role in health and disease [19, 21]. For instance, pseudogenes of cancer-related genes have previously been shown to have regulatory roles in tumor biology [21] and type 2 diabetes [22] by modulating the levels of their corresponding functional gene through posttranscriptional silencing. Our hypotheses are also in agreement with a study on the γ-aminobutyric acid type A (GABA_A_) where alternative splice variants of the GABA_A_ α4 (including one variant that lacks exon 9 which encodes the TM4 domain), are thought to have a posttranslational regulatory role [11, 54]. GABA_A_ receptors are also chloride-gated ion channel receptors playing important roles as inhibitory neurotransmitter receptors in the brain [11, 54, 55].

## Conclusion

The clinical features displayed by DGDP084 are consistent with a neurological dysfunction arising as a result of the Xq22.2 microdeletion. Our comparative deletion mapping and RT-qPCR results support the hypothesis that *GLRA4* is a novel candidate gene for XLID and is likely involved in the intellectual disability, behavioral problems, and craniofacial anomalies seen in DGDP084. Additional point mutations or intragenic deletions of *GLRA4* are required to further substantiate our hypothesis.

## Abbreviations

aCGH, array comparative genomic hybridization; CNS, central nervous system; CNVs, copy number variations; EEG, electroencephalogram; GABA_A_, γ-aminobutyric acid type A; *GAPDH*, glyceraldehyde-3-phosphate dehydrogenase; GLRA1, glycine receptor, alpha-1 subunit; GLRA2, glycine receptor, alpha-2 subunit; GLRA3, glycine receptor, alpha-3 subunit; GLRA4, glycine receptor, alpha-4 subunit; *GLRB*, glycine receptor, beta subunit; GLRs, glycine receptors; *HMGA1*, high mobility group at-hook 1; ID, intellectual disability; *MORF4L2*, mortality factor 4-like protein 2; *MRGX*, morf-related gene x; *PLP1*, proteolipid protein 1; PMD, Pelizaeus-Merzbacher disease; *PTEN*, phosphatase and tensin homolog; *PTENP1*, phosphatase and tensin homolog pseudogene 1; *PTPN11*, protein-tyrosine phosphatase, nonreceptor-type, 11; qPCR, quantitative polymerase chain reaction; *RAB9B*, ras-associated protein 9b; RT-qPCR, reverse-transcriptase quantitative polymerase chain reaction; *TCEAL1*, transcription elongation factor a-like 1; *TCEAL3*, transcription elongation factor a-like 3; TM4, 4th transmembrane domain; XCI, X chromosome inactivation; XLID, X-linked intellectual disability

## Additional file

Additional file 1: Table S1. Primers used for qPCR and RT-qPCR. **Table S2.** Numerical means and standard deviations of qPCR and RT-qPCR on family members. **Table S3.** Numerical means and standard deviations of RT-qPCR on human tissues. (DOCX 17 kb)
