# Supplementary material for: A microdeletion at Xq22.2 implicates a glycine receptor GLRA4 involved in intellectual disability, behavioral problems and craniofacial anomalies
Source: BMC Neurol. 2016 Aug 9;16:132. doi: 10.1186/s12883-016-0642-z (PMC4979147; doi:10.1186/s12883-016-0642-z)
Supplement: Additional file 1: Table S1. — Primers used for qPCR and RT-qPCR. Table S2. Numerical means and standard deviations of qPCR and RT-qPCR on family members. Table S3. Numerical means and standard deviations of RT-qPCR on human tissues. (DOCX 17 kb) [file 12883_2016_642_MOESM1_ESM.docx]

**Additional file 1: Table S1** Primers used for qPCR and RT-qPCR

________________________________________________________________________________________________________

Primer name Forward primer 5’-3’ Reverse primer 5’-3’

________________________________________________________________________________________________________

*#GLRAEx4F1-R1 ACGCCTGTCCTACCGAGAATATC CTCATTAGCAAAGAAGAGGTCTGG

*#MORFAL2Ex1F2-R2 CCAGTATGCTGAAATCCTCTTGGCTC GGCTTTTCTCATCAAGGGGCGTATAG

#TCEAL1Ex1F4-R4 TCCCAACATGGACAAACCACGCAAAGA GGACTGCTCCTCCGAAGACTGC

*Xq22.2F5-R5 TGGATACTATCATATCTGCTGGGC AGAAGGGCTTTGTCACCATGAGTA

*TCEAL15UTRF1-In1R1 AGGAAGAGGCAATACTACCCGAGA CACACCAGGCATTGTCACTCACCT

*Xq22.2F4-R4 ACCCAGCAGAACCAATGGAAGCA AGTACCTCGTCAGCAGACCTTTC

________________________________________________________________________________________________________

* Primers used for qPCR

# Primers used for RT-qPCR

**Additional file 1: Table S2** Numerical means and standard deviations of qPCR and RT-qPCR on family members

________________________________________________________________________________________________________

Individual gene/locus figure

________________________________________________________________________________________________________

Intergenic region1 TCEAL15’-UTR MORF4L2 GLRA4 Intergenic region 2 2

DGDP084 1.11 (0.01) 0.48 (0.08) 0.40 (0.01) 0.54 (0.05) 0.93 (0.08)

Mother 1 (0) 1 (0) 1 (0) 1 (0) 1 (0)

GLRA4 2

DGDP084 0.54 (0.05)

Mother 1 (0)

Father 0.45 (0.27)

GLRA4 MORF4L2 TCEAL1 PLP1 3

DGDP084 0.27 (0.06) 0.87 (0.05) 1.10 (0.11) 1.33 (0.33)

Mother 1 (0) 1 (0) 1 (0) 1 (0)

Father 0.25 (0.02) 1.11 (0.09) 0.98 (0.11) 0.78 (0.3)

________________________________________________________________________________________________________

Mean values are displayed and standard deviations are shown in brackets

**Additional file 1: Table S3** Numerical means and standard deviations of RT-qPCR on human tissues

________________________________________________________________

Tissue gene

_______ _________________________________________________________

GLRA4 MORF4L2 TCEAL1

Brain 26.3 (1.6) 23.95 (0.74) 51.57 (3.7)

Heart 0.24 (0.15) 3.52 (0.6) 7.83 (2.72)

Kidney 0.15 (0.04) 1.41 (0.4) 1.8 (0.02)

Liver 0.17 (0.04) 2.47 (0.07) 1.96 (0.04)

Lung 0.10 (0.02) 0.95 (0.26) 1.0 (0.11)

Skeletal muscle 0.48 (0.24) 9.84 (0.06) 22.42 (3.0)

Lymphocyte 1 (0) 1 (0) 1 (0)

Brain 25.03 (4.6) 23.95 (0.74) 51.57 (3.7)

Fetal brain 58.94 (11.87) 30.84 (0.81) 93.52 (17.17)

Cerebellum 38.09 (4.43) 22.6 (0.26) 84.59 (4.00)

Cerebral cortex 27.78 (6.50) 21.11 (0.14) 48.70 (2.31)

Hippocampus 20.32 (1.04) 19.7 (0.45) 35.92 (0.47)

Lymphocyte 1 (0) 1 (0) 1 (0)

__________________________________________________________________

Mean values are displayed and standard deviations are shown in brackets

All values in Table S3 originate from data presented in Fig. 4
